# Low Vision Profile in Jordan: A Vision Rehabilitation Center-Based Study

**DOI:** 10.3390/healthcare9010020

**Published:** 2020-12-26

**Authors:** Yuser Qutishat, Sami Shublaq, Maisaa Masoud, Nasim Alnuman

**Affiliations:** Vision Rehabilitation Center, The German Jordanian University, Amman 11180, Jordan; sami.shublaq@gju.edu.jo (S.S.); maysa.mitane@gju.edu.jo (M.M.); nasim.alnuman@gju.edu.jo (N.A.)

**Keywords:** vision impairment, ocular pathology, vision rehabilitation, functional visual problems, geographical distributions, referral

## Abstract

The aim of this paper is to study the profile of persons with low vision in Jordan based on the clinical records of service users who attended the Vision Rehabilitation Center (VRC) at the German Jordanian University (GJU). A retrospective study was conducted by reviewing the archived data for persons with low vision attending the VRC over the period September 2012 to December 2017. The information collected included age, gender, referral, geographical distribution, chief functional visual problems, and ocular pathology. The records of 725 (28.9 ± 20.3 years old) persons out of 858 persons were analyzed. Almost half (50.6%) of the sample was less than 18 years old. The main cause of the low vision was retinal diseases (53.4%), followed by albinism. Gender and age showed no significant influence on ocular pathology distribution. For the referrals, ophthalmologists (37.8%) were the largest source of referral, followed by institutions for people with disabilities (14.9%). Near tasks were reported as the main functional problems for patients with low vision (74.9%), followed by distance tasks (8.3%). This study sets a precedent for determining the characteristics of persons with low vision in Jordan. Developing an efficient referral system between eye health care professionals and other health caregivers is important to ensure the best multidisciplinary services for low vision.

## 1. Introduction

According to the World Health Organization (WHO), persons with low vision are those with visual acuity (VA) of ≤6/18 in the better eye, or visual field (VF) <10° after completing medical treatments that include surgical operations, drugs, and the best correction of the refractive error [1]. Based on the WHO World Report of Vision, some eye diseases cause visual impairment, such as age macular degeneration, diabetic retinopathy, and glaucoma [2]. These diseases may affect the execution of daily living activities. Vision rehabilitation presents some solutions in order to overcome such functional problems, such as providing visual aids with training on visual skills to use the residual vision effectively, and advice on how to accommodate the surrounding environment to meet their visual needs. Identification of the functional complaints and evaluation of vision are done by eyecare professionals, either ophthalmologists or optometrists who have special training on vision rehabilitation [3].

Having visual impairment negatively affects the activities of daily life [4]. Low vision rehabilitation services were developed to increase the quality of life for persons with low vision in many aspects, including physical, social, functional, and psychosocial aspects [5,6], through enhancing their participation when meeting their functional needs [7]. Global statistics show that around 70 million people require vision rehabilitation services, but only 5%–10% of those people have access to such services [8]. The unmet needs are attributed to the inconsistent delivery of services between countryside and civilized areas, the low representation of services for people with blindness, the reduced awareness of low vision services among persons with visual impairment, patient mobility difficulties, language obstacles, and poor referrals from eye-health professionals [9]. Another list of barriers mentioned in a study by Chiang, who compared the low vision services in developing and developed countries, included shortage of information available, reduced numbers of eye health professionals, and low vision services that are not well distributed to cover entire countries, especially in the eastern Mediterranean region, where the low vision services vary between no coverage to ≤10%. The last factor to cause fewer services is financial difficulties, where these services are covered by non-governmental organizations [10]. On the other hand, in developed countries such as the Netherlands, persons with visual impairment do not have access to complete services as there is lack of data available on the prevalence and causes of blindness and low vision [11]. In addition, in Canada, ophthalmologists are the core origin of referrals for low vision services, but poor vision does not prompt these referrals for low vision services [12].

Another factor that plays a role in the provision of rehabilitation services for people with low vision is the knowledge of healthcare professionals. A study in India showed that the shortage of specialized services for people with low vision occurred where there was less education, covering vision rehabilitation among ophthalmic health professionals, in addition to the high cost of optical devices [13]. A similar scenario can be seen in Egypt and many other developing countries [14,15]. Vision rehabilitation services should be delivered through a multidisciplinary team to provide a holistic approach for people with low vision; however, this is not the case in many rehabilitation institutions [16]. Not only in developing countries, but also in developed countries, such as Canada, in which the shortage of low vision rehabilitation services is due to the lack of education of optometrists in low-vision assessment, long evaluation times, and the availability of equipment [17]. It is of paramount importance to have specialized rehabilitation services for people with low vision [18,19].

Furthermore, there is shortage of data about rehabilitation service coverage in developing countries. The Universal Health Coverage study, performed on 204 countries [20], including Jordan, a Middle Eastern country, suggested prioritizing data collection in this field. Additionally, many of the available studies are derived from population surveys that lack specific clinical diagnosis [10,20,21].

The Vision Rehabilitation Center (VRC) at the German Jordanian University (GJU) was established in 2012 as a non-profit center located in the capital city. The healthcare services for people with low vision in Jordan were scarce and it is best described as randomized services that lacked the evidence-based approach and included the prescription of optical devices with no rehabilitation. Moreover, there were no guidelines for referrals of persons with low vision to the vision rehabilitation services. The VRC provides its services to people with low vision who have functional problems in their daily life activities through a team of optometrists and therapists specialized in low-vision rehabilitation. The center services include an interview with the service user to take medical history and functional problems; the functional vision assessment; the prescription of magnifying devices; advice for environmental accommodation and compensatory visual strategies.

This study investigates the information and profile of people with low vision in Jordan based on data provided by the VRC over the years 2012–2017. We are particularly interested in clinical measures related to age, gender, referrals, geographical distribution, functional problems, and ocular pathology. These numbers reflect characteristics of people with low vision and may give an overview for better future planning of vision rehabilitation services in Jordan.

## 2. Materials and Methods

A retrospective study was conducted at the VRC in Amman-Jordan. The clinical records of 858 adult and pediatric service users were analyzed. The inclusion criteria were persons with low vision based on the definition of low vision according to the WHO and/or who benefited from the low vision services; referred to the VRC over the period from September 2012 to December 2017; the service users were diagnosed by experienced ophthalmologists and the needed medical intervention was received before they visited the VRC; they were assessed by registered optometrists specialized in low vision; their assessment forms were filled.

The information collected from the service users included the following parts: personal information—gender, age, and address; medical history—the visual impairment as diagnosed by the ophthalmologists; the referral for each service user; chief functional visual problems that occur while executing activities of daily living. The functional problems were addressed by asking the users open questions about the difficulties caused by their visual impairment, and their subjective responses were recorded.

The information collected from the service users were coded and entered on the computer. The statistical tools within MS/Excel (Microsoft Corporation, Redmond, Washington, USA) and MATLAB (r2018b) (MathWorks, Massachusetts, USA) were used to analyze the data. The frequencies of occurrences were reported. The Mann–Whitney test was used to compare whether the distribution of ocular pathologies is the same for the two unpaired age groups and gender groups, and the results were considered statistically significant when the *p*–value was <0.05. The study was conducted in compliance with the declaration of Helsinki and was approved by the research ethics committee at the GJU, approval number (REC/1/2020).

## 3. Results

Out of the 858 first-time visitors to the VRC during the six-year study period, 133 subjects were excluded as they did not meet the inclusion criteria. The remaining 725 service users consisted of 698 low-vision subjects and 27 subjects with no low vision but who benefited from the low-vision services and received assistive devices.

### 3.1. Age and Gender

**Figure 1:**
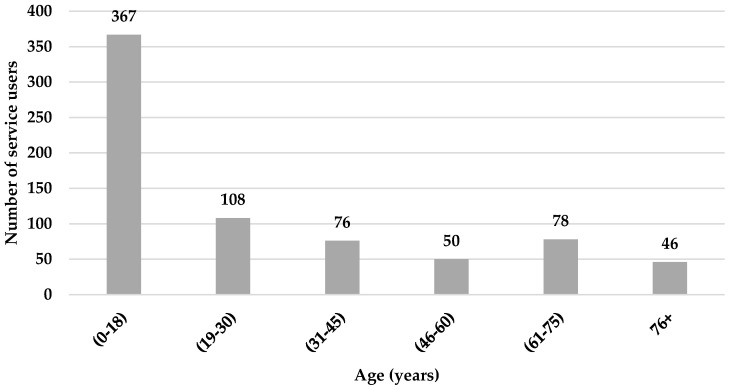
Age distribution of service users.

The 725 service users’ ages ranged between 3 months and 92 years with a mean age of 28.9 (±20.3) years. The age distribution of the service users is shown in Figure 1. Out of the 725 subjects, 367 (50.6%) were children below 18 years old, which was the largest age group proportion, followed by the 19–30 age group (14.9%). The percentage of males was higher than females, 410 (56.6%) and 315 (43.4%), respectively.

### 3.2. Referral and Geographical Distribution

**Figure 2:**
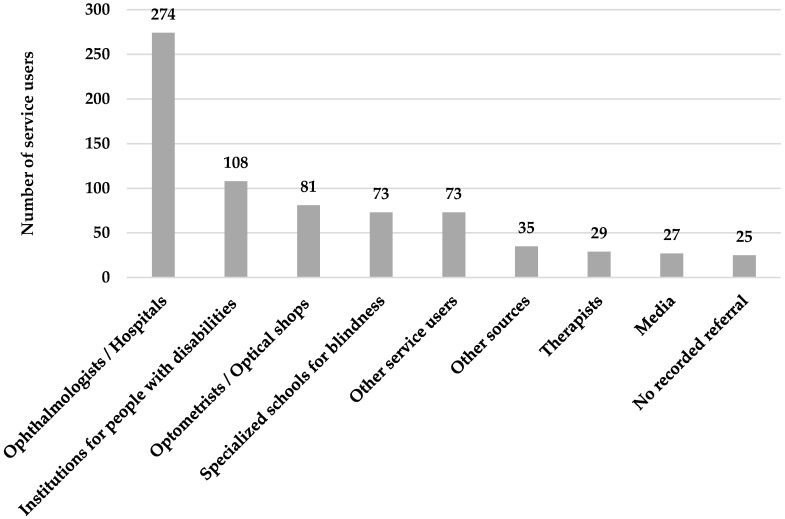
Referral sources of service users.

The highest percentage of service users, 37.8% (*n* = 274), was referred from ophthalmologists and hospitals that had ophthalmology departments, followed by referrals from institutions for people with disabilities with 14.9% (*n* =108). Eighty-one service users (11.2%) were from optometrists and optical shops, 10.1% (*n* = 73) were from specialized schools for blindness, and 10.1% (*n* = 73) were from other service users who visited the center previously. The rest of the referrals varied between 3.4% to 4.8%. Institutions for people with disabilities include organizations and centers dealing with persons with different physical and mental disabilities associated with visual impairment. Other sources include friends, relatives and colleagues. Therapists include occupational and physical therapists. Figure 2 presents the referral sources of the patients.

According to the 2017 census, the population of Jordan was 10,053,000. The highest population, 42.0% of the total, is centralized in the capital city (Amman), followed by 18.6% in Irbid (north), then 14.2% in Zarqa (north-east). The cities in the south of Jordan contain only 7.8% of the whole population [22]. Geographically, the majority of the service users referred to the VRC were from Amman, comprising 62.5% of the total; 14.5% were from the north-east of Jordan, 8.8% were from the northern parts of Jordan, 6.6% were from the north-west of Jordan, and 3.2% were from the south of Jordan. The lowest percentages of service users were from other countries (3.0%) and from Syrian refugee camps (1.4%). Table 1 presents the geographical distribution of the service users.

### 3.3. Main and Secondary Functional Problems

**Figure 3:**
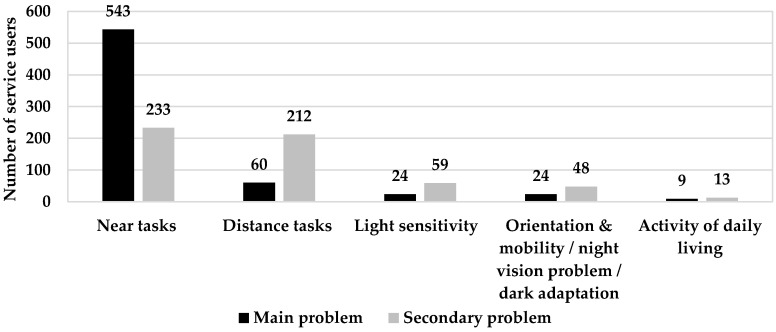
Main and secondary functional problems of service users. Near tasks include reading, writing, work tasks, and technology use. Distance tasks include watching TV, seeing the class board, seeing signs, face recognition, and driving. Light sensitivity includes glare, photophobia, and sun sensitivity. The numbers do not sum up to the sample size (725) since 65 subjects did not report any main or secondary functional problem and 95 subjects did not report any secondary functional problem.

Main and secondary functional problems are shown in Figure 3. Near tasks (reading, writing, work tasks and technology use) were reported as the vast majority of main function problems by the service users (74.9%), while distance tasks, which include watching TV, seeing the class board, seeing signs, faces recognition, and driving, formed only 8.3%. In secondary functional problems, near and distance tasks were very close together, including 32.1% and 29.2% of reports, respectively. Of the 725 assessed subjects, 65 subjects did not report any main or secondary functional problem, and 95 subjects reported only one functional problem.

### 3.4. Ocular Pathology

The main frequent cause of low vision among our service users was retinal diseases, reported by 387 patients (53.4%). Retinal diseases were the most frequent cause in both age groups, ≤18 (46%) and >18 years (60.6%), then albinism (8.8%). The distributions of the optic nerve diseases and other ocular diseases were 7.2% and 6.5%, respectively, glaucoma (6.5%), multiple physical and mental disabilities associated with visual impairment (2.8%). These disabilities include Cerebral Palsy, Laurence Moon Biedl syndrome, Joubert syndrome, Usher syndrome, Peter’s Anomaly, Cortical Visual Impairment and Alstrom syndrome. The other ocular diseases included high myopia, keratoconus, corneal opacity, microphthalmy, and others. The remaining group (11%) represents the service users with unknown diagnosis because they did not have their medical report available when they visited the VRC. The causes of low vision, according to age and gender, are presented in Table 2. The distribution of the common ocular pathologies was not significantly related to the age (age ≤ 18 years *n* = 367, age > 18 years *n* = 358, *p* = 0.509, Mann–Whitney test) or the gender within each age group (male *n* = 196, female *n* = 171, *p* = 0.704 (within age group ≤ 18 years); male *n* = 214, female *n* = 144, *p* = 0.412 (within age group > 18 years), Mann–Whitney test). By dividing the adults’ group into two age groups: first group (18–44 years) and second group (45+ years), the distribution of the common ocular pathologies also showed no significant relation (age 18–44 years *n* = 183, age ≥ 45 years *n* = 175, *p* = 0.810, Mann–Whitney test).

There were 10 types of retinal diseases recorded in the VRC, with the highest frequency of occurrence for retinitis pigmentosa (*n* = 95, 24.5%), followed by macular dystrophies (*n* = 48, 12.4%), and retinal dystrophies (*n* = 48, 12.4%), while the lowest frequency of occurrence was for achromatopsia (*n* = 7, 1.8%). The detailed distribution of the types of retinal disease frequencies of occurrence are shown in Table 3.

## 4. Discussion

The data derived from records of low-vision clinics have advantages over surveys or registration as it gives more detailed and representative information regarding the characteristics of the service users and the provided services [23,24]. In this study, data are presented from the records of service users who visited the VRC from its opening in September 2012 until December 2017.

From the whole sample that visited the VRC and benefited from its services, children (0–18 years) were the largest group, with 50.6% of total users, followed by the next age group (19–30 years) with 14.9%. In an Indian study, they found that 45.46% of the sample was in the age range of 10–29 years [3]. A study from Egypt also found that 46% of the patients enrolled in their study were between the ages 6–40 years old [14]. Another study in Nigeria found that 19.2% of patients were between 10–19 years old [21]. In addition to the mentioned studies from developing countries, further studies in Korea [25], Malaysia [26], and Nepal [27,28,29] found that the highest percentage of service users were from younger age groups.

The high number of the children in our study can be explained by the fact that the patients are of school age, where parents and teachers pay attention to the students’ academic functioning, especially in reading and seeing the class board. Additionally, the young age distribution is consistent with the estimated population of Jordan by age group at the end of 2017, which determined that 62.9% of the Jordanian population was 0–29 years old, 31.7% was 30–59 years, and 5.5% was over 60 years old [22]. On the other hand, other reports from developed countries found that the highest age proportion was for older service users above 60 years old [23,24,30,31,32].

This difference between developed and developing countries can also be related to the lower literacy rate for older patients in the developing countries [33] and, as a result, they will have less interest in using their residual vision for reading. On the other hand, older patients in developing countries, as part of their culture, are not living alone but usually with their extended families and, therefore, they are given assistance by their caregivers/family members and may find assistance to be easier than using assistive devices and life accommodations. Gold suggests that a lack of awareness and understanding of vision loss is one of the barriers to the use of low vision services, as older people often accept their vision deterioration as a natural part of ageing [12].

In this study, the percentage of males was higher than females (56.6%, 43.4%). All the previously mentioned studies that were conducted in the developing countries also had high male to female ratios. This can be explained due to the low usage of eye care services and lower acceptance of treatment by females in developing countries [34,35]. The population of Jordan is 10,053,000, based on the 2017 census, and 47.1% (4,730,000) are females and 52.9% (5,323,000) are males [22].

Ophthalmologists are the first level of contact with the patients, and they play a key role in the referral process. They can be facilitators or barriers for people with low vision to benefit from the available services [17]. The major source for referral to the VRC was ophthalmologists in hospitals and eye clinics, which was consistent with the Canadian and Australian studies [12,36]. In another Australian study, Jamous further concluded that ophthalmologists mainly referred patients to low-vision rehabilitation clinics, while optometrists tended to refer most of their patients to the ophthalmologists themselves [37]. An additional study from low-vision clinics in the eastern region of Nepal found that 87.98% of the cases were referred from the eye-care professionals, while 8.66% were referred from schoolteachers and 2.71% were referred from community-based rehabilitation (CBR) [27].

These results show the basic role of the eye-care professionals and the importance of the communication and referrals between them, as many studies mentioned that the miscommunication between eye-care professionals is a crucial barrier for referral [9,12,17,38,39,40,41,42]. Additionally, poor referral criteria and the lack of information about the available services are considered to be major barriers to benefiting from vision rehabilitation services [17,43].

Regarding geographical distributions, most of the service users were from the capital Amman (Urban Area). This can be explained by several factors and barriers that are discussed in many studies; people with low vision from outside Amman are living far away and they need time, transportation, and money to visit the VRC. In addition, the awareness of services is low and many people with vision impairment do not understand what vision rehabilitation requires, especially in rural areas [9,10,12,17,38,44,45,46].

Out of the whole sample of the service users, 74.9% had main functional problems with near tasks, mainly reading. Many other studies reported that most of the people with low vision considered reading as the most common functional problem, such as Owsley [47] (85.9%), Brown [48] (66.4%), Elliot [23] (75%), and Kim [25] (60.8%). Shaaban reported that 54% of patients asked for near devices and 34% asked for aids to help in both near and distance tasks [12]. Many studies reported that the most commonly prescribed low-vision devices were for near tasks [3,24,26,28,29,31,32]. On the other hand, reading a textbook at arm’s length, copying from the blackboard, seeing somebody across the road, and identifying colors were the four most difficult tasks in an Egyptian study [49].

The results regarding ocular pathology showed that retinal diseases were the main cause of issues, affecting 53.4% from the whole population. Retinitis pigmentosa was the most common issue within retinal diseases, affecting 24.5% of patients. This is compatible with a Jordanian study that discussed the causes of severe visual impairment and blindness and found that retinitis pigmentosa was the most common cause of severe visual impairment among adults (29.7%), followed by diabetic retinopathy (19.9%) and glaucoma (15.8%) [50]. A study done by Mohidin and Yusof found that retinitis pigmentosa caused the highest percentage of the ocular pathologies for patients aged between 30–59 years old, followed by macular dystrophy and diabetic retinopathy [26]. Another study found that retinitis pigmentosa was the most common cause of low vision in the age group 15–60 years and was the second most common cause in the whole population after refractive errors and amblyopia [27]. A third study stated that 16.6% of ocular issues were caused by retinitis pigmentosa, followed by age-related macular degeneration, then albinism [21].

Several studies showed that retinal diseases are the main reason for visual impairment [3,10,28,29,47,51]. In contrast, other studies revealed that uncorrected refractive errors are the main cause of visual impairment [52,53,54], which is similar to global data from the WHO, which mentioned that 43% of the causes of visual impairment are due to uncorrected refractive errors [1]. In our study, congenital and hereditary diseases were shown to occur more frequently than preventable diseases. This can be explained by the high number of consanguineous marriages that are very common in Jordan and other Arab countries, such as West Bank and Yemen [55,56]. In addition, the younger age groups in our study (51% less than 18 years old) play a major role in identifying more congenital and hereditary diseases.

In this study, retinal diseases were the main cause of low vision for children under 18 years old. A study in Pakistan found that the leading ocular pathologies that caused low vision for children aged 4–16 years old were retinal diseases (32%), nystagmus (15%), and oculocutaneous albinism (7%) [57]. Retinal diseases (52%) were also the main cause of low vision in West Bank and Gaza, followed by optic atrophy (12%) and glaucoma (9%) [55]. In India, it was recorded that hereditary macular degeneration and retinitis pigmentosa were the second most common causes of low vision for children, after congenital glaucoma [13]. Albinism is among the diseases that cause the highest percentages of low vision for children in Egypt (44%) and in Nigeria (24.4%) [21,49]. In Yemen and Nepal, refractive errors and amblyopia were the major causes of low vision at 29.2% and 30.33%, respectively [27,56]. When comparing the distribution of common ocular pathologies as dependent variables between the different age groups (age ≤ 18 years and age > 18 years), no significant relations were found (*p* = 0.509).

Eye diseases, such as cataracts, corneal ulceration, and infections, represent the dominant diseases that cause low vision in some developing countries [58,59,60]. These diseases are less present as causes of low vision in Jordan. This is probably because of the relative improvements in health care access and delivery and post-natal medical follow ups, which lead to a higher probability of survival for preterm and newborn children [59] and due to the early treatment of those preventable causes [25,61].

Multiple physical and mental disabilities associated with visual impairment are the least-common cases for low vision in this study sample, which was not the case in two studies in the Netherlands where the cerebral visual impairments (CVI) caused a high percentage of low-vision cases, and they interpret these results as being due to the increased survival of preterm and low-birth-weight children and improved diagnostic possibilities [7,11]. Gilbert and Foster reported that prenatal conditions, such as lesions of the central nervous system, are more common in high-income economies, and acquired conditions are more common in low-income countries [62] and this may explain the low number of CVI cases in this study in Jordan.

## 5. Conclusions

In conclusion, near tasks, including reading, writing and the use of mobile phones, were the main visual functional needs for people with low vision. The leading cause of low vision in the study sample was retinal diseases, with retinitis pigmentosa having the highest distribution among the studied retinal diseases. This suggests increasing community awareness regarding hereditary diseases, especially those related to consanguineous marriages. Children with low vision represent half of the sample population, which emphasize schools’ crucial role in identifying students with low vision and referring them to vision rehabilitation services.

The highest referrals rate for low vision services was from ophthalmologists. The development of an effective referral system between the different healthcare professionals and vision rehabilitation specialists is necessary to provide efficient multidisciplinary services. Most of service users came from the capital city and other urban areas and fewer users came from rural places. Raising awareness about the existence of services for low vision among the country and opening specialized centers distributed over wider geographical areas are two challenges to be addressed by the state and policy makers.

## Figures and Tables

**Table 1:** Geographical distribution of service users.

Region	No.	%
Amman (Capital)	453	62.5
North-eastern Jordan	105	14.5
North Jordan	64	8.8
North-western Jordan	48	6.6
South Jordan	23	3.2
From Other Countries	22	3.0
Syrian Refugee camps	10	1.4
Total	725	100

**Table 2:** Distribution of age and gender of the service users based on their ocular pathology.

Ocular Pathology	Ages (Years)								Total	
	≤18				>18					
	Males		Females		Males		Females			
	No.	%	No.	%	No.	%	No.	%	No.	%
Retinal diseases	85	43.4	83	48.5	136	63.6	83	57.6	387	53.4
No medical diagnosis	31	15.8	18	10.5	14	6.5	17	11.8	80	11
Albinism	25	12.8	21	12.3	12	5.5	6	4.2	64	8.8
Optic nerve diseases	12	6.1	12	7.0	15	7.0	13	9.0	52	7.2
Other ocular diseases	14	7.1	13	7.6	13	6.1	7	4.9	47	6.5
Glaucoma	13	6.6	10	5.8	12	5.6	12	8.3	47	6.5
Cataract	6	3.1	6	3.5	11	5.1	5	3.5	28	3.9
Multiple disabilities associated with visual impairment	10	5.1	8	4.6	1	0.5	1	0.7	20	2.8
Total	196	100	171	100	214	100	144	100	725	100
*p*-value	0.704				0.412					
	0.509									

**Table 3:** Frequencies of occurrence for different types of retinal diseases.

Types of Retinal Diseases	No.	%
Retinitis pigmentosa	95	24.5
Macular dystrophies	48	12.4
Retinal dystrophies	48	12.4
Age macular degeneration	40	10.3
Stargardt’s disease	38	9.8
Diabetic retinopathy	36	9.3
Rod-cone dystrophy	31	8.0
Retinal detachment	24	6.2
Retinopathy of prematurity	20	5.2
Achromatopsia	7	1.8
Total	387	100

## Data Availability

The data presented in this study are available on request from the corresponding author after university approval. The data are not publicly available due to privacy or ethical restrictions.

## References

[1] World Health Organization (2010). Global Data on Visual Impairments.

[2] World Health Organization (2019). World Report on Vision.

[3] Verma K.M., Tondon M. (2015). Evaluation of low vision and its rehabilitation in various disorders. Indian J. Clin. Exp. Ophthalmol..

[4] Haymes S.A., Johnston A.W., Heyes A.D. (2002). Relationship between vision impairment and ability to perform activities of daily living. Ophthalmic Physiol. Opt..

[5] Stelmack J. (2001). Quality of Life of Low-Vision Patients and Outcomes of Low-Vision Rehabilitation. Optom. Vis. Sci..

[6] Draper E.M., Feng R., Appel S.D., Graboyes M., Engle E., Ciner E.B., Ellenberg J.H., Stambolian D. (2016). Low Vision Rehabilitation for Adult African Americans in Two Settings. Optom. Vis. Sci..

[7] Rainey L., Nispen R.V., Rens G.V. (2013). Evaluating rehabilitation goals of visually impaired children in multidisciplinary care according to ICF-CY guidelines. Acta Ophthalmol..

[8] Chiang P.P.-C. (2009). The Global Mapping of Low Vision Services. Ph.D. Thesis.

[9] O’connor P.M., Mu L.C., Keeffe J.E. (2008). Access and utilization of a new low-vision rehabilitation service. Clin. Exp. Ophthalmol..

[10] Chiang P.P.-C., O’Connor P.M., Mesurier R.T.L., Keeffe J.E. (2011). A Global Survey of Low Vision Service Provision. Ophthalmic Epidemiol..

[11] Boonstra N., Limburg H., Tijmes N., Genderen M.V., Schuil J., Nispen R.V. (2011). Changes in causes of low vision between 1988 and 2009 in a Dutch population of children. Acta Ophthalmol..

[12] Gold D., Zuvela B., Hodge W.G. (2006). Perspectives on low vision service in Canada: A pilot study. Can. J. Ophthalmol..

[13] Gothwal V. (2000). Characteristics of a paediatric low vision population in a private eye hospital in India. Ophthalmic Physiol. Opt..

[14] Shaaban S., El-Lakkany A., Swelam A., Anwar G. (2009). Low vision Aids provision for visually impaired Egyptian patients-a clinical outcome. Middle East Afr. J. Ophthalmol..

[15] Singh S.S. (2017). Bridging the Gap between Medical Low Vision and Visual Rehabilitation Services in Developing Nations. Delhi J. Ophthalmol..

[16] Dickinson C., Linck P., Tudor-Edwards R., Binns A., Bunce C., Harper R., Jackson J., Lindsay J., Suttie A., Wolffsohn J. (2011). A profile of low vision services in England: The Low Vision Service Model Evaluation (LOVSME) project. Eye.

[17] Lam N., Leat S.J., Leung A. (2015). Low-vision Service Provision by Optometrists. Optom. Vis. Sci..

[18] Wang B.Z., Pesudovs K., Keane M.C., Daly A., Chen C.S. (2012). Evaluating the Effectiveness of Multidisciplinary Low-Vision Rehabilitation. Optom. Vis. Sci..

[19] Leat S.J. (2016). A Proposed Model for Integrated Low-Vision Rehabilitation Services in Canada. Optom. Vis. Sci..

[20] Lozano R., Fullman N., Mumford J.E., Knight M., Barthelemy C.M., Abbafati C., Abbastabar H., Abd-Allah F., Abdollahi M., Abedi A. (2020). Measuring universal health coverage based on an index of effective coverage of health services in 204 countries and territories, 1990–2019: A systematic analysis for the Global Burden of Disease Study 2019. Lancet.

[21] Olusanya B., Onoja G., Ibraheem W., Bekibele C. (2012). Profile of patients presenting at a low vision clinic in a developing country. BMC Ophthalmol..

[22] Jordan Statistical Yearbook 2017. Department of Statistics Web Site. http://dosweb.dos.gov.jo/products/statistical_yearbook2017/.

[23] Elliott D.B., Trukolo-Ilic M., Strong J.G., Pace R., Plotkin A., Bevers P. (1997). Demographic characteristics of the vision-disabled elderly. Investig. Ophthalmol. Vis. Sci..

[24] Wolffsohn J.S., Cochrane A.L. (1999). The Changing Face of the Visually Impaired: The Kooyong Low Vision Clinic’s Past, Present, and Future. Optom. Vis. Sci..

[25] Kim J.H., Joo K.S., Moon N.J. (2010). Characteristics of 681 Low Vision Patients in Korea. J. Korean Med. Sci..

[26] Mohidin N., Yusoff S. (1998). Profile of a low vision clinic population. Clin. Exp. Optom..

[27] Thakur A.K., Joshi P., Kandel H., Bhatta S. (2011). Profile of low vision clinics in eastern region of Nepal. Br. J. Vis. Impair..

[28] Gyawali R., Paudel N., Adhikari P. (2012). Quality of life in Nepalese patients with low vision and the impact of low vision services. J. Optom..

[29] Paudel P., Khadka J., Sharma A. (2005). Profile of a low vision population. Int. Congr. Ser..

[30] Leat S. (1990). The experience of a university based low vision clinic. Ophthalmic Physiol. Opt..

[31] Shuttleworth G.N., Dunlop A., Collins J.K., James C.R. (1995). How effective is an integrated approach to low vision rehabilitation? Two year follow up results from south Devon. Br. J. Ophthalmol..

[32] Lindsay J., Bickerstaff D., Mcglade A., Toner A., Jackson A.J. (2004). Low vision service delivery: An audit of newly developed outreach clinics in Northern Ireland*. Ophthalmic Physiol. Opt..

[33] Hammoud H.R. (2005). Illiteracy in the Arab World. Paper Commissioned for the EFA Global Monitoring Report 2006, Literacy for Life, UNESCO. https://unesdoc.unesco.org/ark:/48223/pf0000146282.

[34] Fletcher A.E. (1999). Low Uptake of Eye Services in Rural India. Arch. Ophthalmol..

[35] Snellingen T., Shrestha B.R., Gharti M.P., Shrestha J.K., Upadhyay M.P., Pokhrel R.P. (1998). Socioeconomic barriers to cataract surgery in Nepal: The south Asian cataract management study. Br. J. Ophthalmol..

[36] Keeffe J.E., Lovie-Kitchin J.E., Taylor H.R. (1996). Referral to low vision services by ophthalmologists. Aust. N. Z. J. Ophthalmol..

[37] Jamous K.F., Jalbert I., Kalloniatis M., Boon M.Y. (2013). Australian optometric and ophthalmologic referral pathways for people with age-related macular degeneration, diabetic retinopathy and glaucoma. Clin. Exp. Optom..

[38] Spafford M.M., Rudman D.L., Leipert B.D., Klinger L., Huot S. (2009). When Self-Presentation Trumps Access: Why Older Adults with Low Vision Go Without Low-Vision Services. J. Appl. Gerontol..

[39] Pollard T.L., Simpson J.A., Lamoureux E.L., Keeffe J.E. (2003). Barriers to accessing low vision services. Ophthalmic Physiol. Opt..

[40] Maclachlan J., Rudman D.L., Klinger L. (2007). Low Vision: A Preliminary Exploration of Its Impact on the Daily Lives of Older Women and Perceived Constraints to Service Use. Phys. Occup. Ther. Geriatr..

[41] Southall K., Wittich W. (2012). Barriers to Low Vision Rehabilitation: A Qualitative Approach. J. Vis. Impair. Blind..

[42] Overbury O., Wittich W. (2011). Barriers to Low Vision Rehabilitation: The Montreal Barriers Study. Investig. Opthalmol. Vis. Sci..

[43] Adam R., Pickering D. (2007). Where Are All the Clients? Barriers to Referral for Low Vision Rehabilitation. Vis. Impair. Res..

[44] Mwilambwe A., Wittich W., Freeman E.E. (2009). Disparities in awareness and use of low-vision rehabilitation. Can. J. Ophthalmol..

[45] Gold D., Simson H. (2005). Identifying the needs of people in Canada who are blind or visually impaired: Preliminary results of a nation-wide study. Int. Congr. Ser..

[46] Walter C., Althouse R., Humble H., Leys M., Odom J. (2004). West Virginia survey of visual health: Low vision and barriers to access. Vis. Impair. Res..

[47] Owsley C. (2009). Characteristics of Low-Vision Rehabilitation Services in the United States. Arch. Ophthalmol..

[48] Brown J.C., Goldstein J.E., Chan T.L., Massof R., Ramulu P. (2014). Characterizing Functional Complaints in Patients Seeking Outpatient Low-Vision Services in the United States. Ophthalmology.

[49] Mousa A., El Byoumi M.B. (2010). Visual Function of Egyptian Children with Low Vision and the Demographic Determinants. Middle East Afr. J. Ophthalmol..

[50] Baarah B., Shatnawi R., Khatatbeh A. (2018). Causes of permanent severe visual impairment and blindness among Jordanian population. Middle East Afr. J. Ophthalmol..

[51] Pardhan S., Mahomed I. (2002). The clinical characteristics of Asian and Caucasian patients on Bradford’s Low Vision Register. Eye.

[52] Wong T.Y. (2008). Prevalence and Causes of Low Vision and Blindness in an Urban Malay Population. Arch. Ophthalmol..

[53] Bourne R.R.A., Jonas J.B., Flaxman S.R., Keeffe J., Leasher J., Naidoo K., Parodi M.B., Pesudovs K., Price H., White R.A. (2014). Prevalence and causes of vision loss in high-income countries and in Eastern and Central Europe: 1990–2010. Br. J. Ophthalmol..

[54] Keeffe J., Taylor H.R., Fotis K., Pesudovs K., Flaxman S.R., Jonas J.B., Leasher J., Naidoo K., Price H., White R.A. (2014). Prevalence and causes of vision loss in Southeast Asia and Oceania: 1990–2010. Br. J. Ophthalmol..

[55] Elder M.J., Cock R.D. (1993). Childhood blindness in the West Bank and Gaza strip: Prevalence, aetiology and hereditary factors. Eye.

[56] Bamashmus M., Akily S.A. (2010). Profile of childhood blindness and low vision in Yemen: A hospital-based study. East. Mediterr. Health J..

[57] Shah M., Khan M., Khan M.T., Khan M.Y., Saeed N. (2011). Causes of visual impairment in children with low vision. J. Coll. Phys. Surg. Pak..

[58] Waddell K.M. (1998). Childhood blindness and low vision in Uganda. Eye.

[59] Khandekar R., Kishore H., Mansu R., Awan H. (2014). The Status of Childhood Blindness and Functional Low Vision in the Eastern Mediterranean Region in 2012. Middle East Afr. J. Ophthalmol..

[60] Durnian J.M., Cheeseman R., Kumar A., Raja V., Newman W., Chandna A. (2009). Childhood sight impairment: A 10-year picture. Eye.

[61] Maida J.M., Mathers K., Alley C.L. (2008). Pediatric ophthalmology in the developing world. Curr. Opin. Ophthalmol..

[62] Gilbert C., Foster A. (2001). Childhood blindness in the context of VISION 2020-the right to sight. Bull. World Health Organ..

